# Design of a Capacitance-to-Digital Converter Based on Iterative Delay-Chain Discharge in 180 nm CMOS Technology

**DOI:** 10.3390/s22010121

**Published:** 2021-12-24

**Authors:** Mattia Cicalini, Massimo Piotto, Paolo Bruschi, Michele Dei

**Affiliations:** Department of Information Engineering, University of Pisa, 56122 Pisa, Italy; mattia.cicalini@phd.unipi.it (M.C.); massimo.piotto@unipi.it (M.P.); paolo.bruschi@unipi.it (P.B.)

**Keywords:** capacitance-to-digital converter, iterative-delay-chain discharge, CMOS capacitive sensor interface

## Abstract

The design of advanced miniaturized ultra-low power interfaces for sensors is extremely important for energy-constrained monitoring applications, such as wearable, ingestible and implantable devices used in the health and medical field. Capacitive sensors, together with their correspondent digital-output readout interfaces, make no exception. Here, we analyse and design a capacitance-to-digital converter, based on the recently introduced iterative delay-chain discharge architecture, showing the circuit inner operating principles and the correspondent design trade-offs. A complete design case, implemented in a commercial 180 nm CMOS process, operating at 0.9 V supply for a 0–250 pF input capacitance range, is presented. The circuit, tested by means of detailed electrical simulations, shows ultra-low energy consumption (≤1.884 nJ/conversion), excellent linearity (linearity error 15.26 ppm), good robustness against process and temperature corners (conversion gain sensitivity to process corners variation of 114.0 ppm and maximum temperature sensitivity of 81.9 ppm/°C in the −40 °C, +125 °C interval) and medium-low resolution of 10.3 effective number of bits, while using only 0.0192 mm${}^{2}$ of silicon area and employing 2.93 ms for a single conversion.

## 1. Introduction

Capacitive sensing technologies underpin many sensory applications, including industrial, automotive, consumer [1] and life-science electronics [2]. At the same time, dedicated and power-optimized readout interfaces have been proposed to take full advantage of this technology. In this sense, capacitance-to-digital converters (CDCs) represent a class of integrated interfaces capable of delivering a digital output readout of the capacitive sensor. Many architectures of CDCs are demonstrated in the literature, exploiting the principles of phase/pulse modulation (PM) [3,4,5,6,7], ΔΣ modulation (ΔΣM) [8,9,10] and capacitive successive approximation register (CSAR) [11,12,13,14,15]. A detailed review of these techniques can be found in [16].

Recently, a simple and compact solution, which presents a significant number of innovations over other kinds of CDCs, was proposed in [17]. The most relevant innovations regard that (i) the CDC implementation is based on basic digital gates (inverters, Nands and Xors); (ii) an external clock signal is not required; and, (iii) as it will be clear in the remainder of this paper, the scaling of the capacitance full scale, i.e., the maximum capacitance value that can be converted, does not affect the internal state variables range in terms of voltage headroom and/or current intensity, as it usually occurs in many other CDC architectures. This fact allows for the extension of the CDC dynamic range (DR) relying only on the length extension of the digital output register. However, the inner working principles of the iterative delay-chain discharge (IDCD) architecture are poorly explained, leaving the designer with numerous unknowns hindering the adoption of this architecture despite its excellent performance in terms of power.

In this work, we address this issue by providing a deeper insight into this new architecture by giving a formal (rather than heuristic) explanation of the CDC operating principle. This discloses the CDC’s intrinsic limits, thus providing awareness of the fundamental trade-offs. Moreover, the analysis paves the way for different implementations of the same architecture that better adapts to specific cases within the voltage-headroom/signal-bandwidth design space.

**Figure 1:**
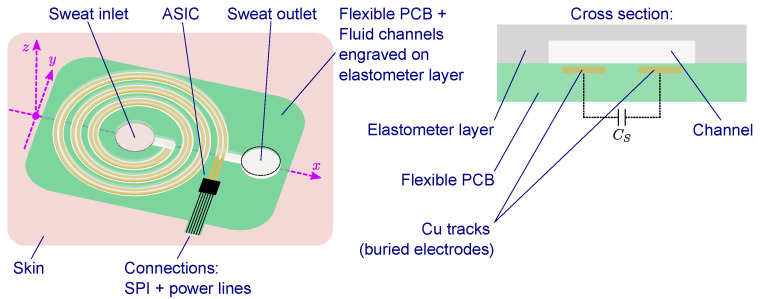
Concept of a wearable platform for volumetric sweat-rate sensing.

The target capacitive sensor considered in this work derives from the wearable platform for sweat-rate sensing sketched in Figure 1. This device is intended to be used for activity tracking in sport applications, and it consists of (i) a flexible printed-circuit board (FPCB) layer, typically a polyimide film; (ii) a decorated elastometer layer, typically polydimethylsiloxane (PDMS), and (iii) an application-specific integrated circuit (ASIC) [18,19,20,21]. The fluidic pathway is then formed by sealing the two layers together and providing an inlet and an outlet, facing, respectively, the skin and the air. In correspondence to the fluidic pathway, two buried electrodes, implemented by the FPCB Cu tracks, work as electrostatically coupling electrodes, providing the capacitive transduction mechanism for the volume occupied by the sweat within the channel. By taking successive capacitance measurements, the volumetric sweat flow can be reconstructed. The measurement readout control is provided by the ASIC, which is placed in close proximity to the sensor in order to avoid interference and excessive parasitic coupling. The ASIC may also provide a standard digital interface, e.g., a serial peripheral interface (SPI), for communication with an external wireless communication module. Preliminary estimation of the capacitance range of structures, such as those in Figure 1, suggests values between 10 and 250 pF, depending on the specific channel geometries and constitutive materials. Similar capacitance range can also be found in other capacitive sensors [22,23].

A 0–250 pF capacitive sensor interface, applying the design rules resulting from the theoretical analysis, is implemented in the UMC 180 nm complementary metal–oxide–semiconductor (CMOS) technology. The chosen capacitive conversion range is compatible with a number of micro-electro-mechanical systems (MEMS) capacitive sensors. Detailed electrical simulations show the following converter performance: systematic input offset of 255.6 fF, linearity error of 15.26 ppm, worst-case process-corner sensitivity on the conversion gain of 114 ppm, temperature sensitivity of 81.9 ppm/°C, maximum signal-to-noise ratio (SNR) of 63.9 dB and maximum conversion energy of 1.884 nJ when operated at 0.9 V supply. In the discussion section of this work, these figures are compared to those of [17] in order to provide insight into the porting of this architecture across different CMOS technological nodes.

## 2. Materials and Methods

Electrical simulations were performed on a 3.3 GHz 14 core CPU x86-64 workstation, operated through CentOS 7, and Cadence IC6.1.7 (ADEXL, Spectre simulator and AMS simulator). The CMOS design kit from UMC 180 nm mixed mode/RF was made available from the Europractice IC Service to European academic and research institutions. Graphical data preparation and presentation were performed by means of Python 3.5.2 importing the following modules: Numpy 1.17.0 and Matplotlib 3.0.3.

## 3. Results

The CDC operation principle is analysed for the first time in Section 3.1, while its implementation in the commercial 180 nm CMOS technology is presented in Section 3.2, followed by detailed electrical simulation in Section 3.3.

### 3.1. Principle of Operation

**Figure 2:**
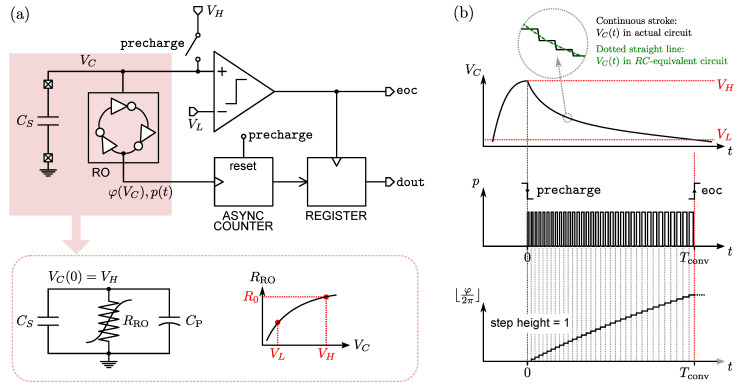
Simplified CDC operation based on a voltage level comparator: (**a**) block-level schematic diagram comprising an RC-circuit equivalent of the RO; (**b**) chronograms of the most important signals.

The CDC operation consists of the discharge of the capacitance $C_{S}$ between two voltage levels, $V_{H}$ and $V_{L}$, with $V_{H}$ being the precharge value and $V_{L}$ the value assumed at the end of the conversion (see Figure 2). For the sake of a clearer explanation, let us assume that $C_{S}$ has one of its terminals connected to the ground. The conversion operation starts by the falling edge of the precharge signal. The discharging of $C_{S}$ supplies the attached ring oscillator (RO), simply implemented by inverter gates, which starts oscillating at a frequency determined by its supply voltage ($V_{C}$). The output of the RO is the frequency modulated two-level signal p(t), whose instantaneous oscillation frequency encodes the amplitude $V_{C}$. The integral of this quantity is the phase φ, which is updated at every cycle as shown in Figure 2b. The oscillation frequency decreases by decreasing $V_{C}$ since the overdrive voltages of the logical gates are decreasing, thus slowing the charge of the next gate in the ring.

While the oscillation edge completes a loop, i.e., φ completes a full cycle, the RO absorbs a certain amount of charge from $C_{S}$, which causes $V_{C}$ to decrease in time. An asynchronous counter keeps track of the number of loops. Finally, $V_{C}$ reaches the $V_{L}$ level, eventually detected by a voltage comparator set to the $V_{L}$ threshold, which, in turn, produces the end-of-conversion signal (eoc) used also to strobe the counter value (dout) into an output register.

Since each loop consumes a certain quantity of charge q[i] (at *i*-th loop), the following relationship must hold:

(1) $$\sum_{i=1}^{N}q\left[i\right]+q_{\epsilon }=C_{S}\left(V_{H}-V_{L}\right),$$

where *N* is the number of loops during the discharge, and $q_{\epsilon }$ is the residual error due to the last incomplete loop.

The heuristic conclusion drawn in [17] is that *N* is proportional to $C_{S}$ apart from the quantization error $q_{\epsilon }/\left(V_{H}-V_{L}\right)$. Nevertheless, Equation (1) does not give any support to this conclusion since the relation between *N* and $C_{S}$ is not explicit. Moreover, since the RO supply voltage-to-frequency characteristic is generally non-linear, the capacitance-to-digital conversion law is not evident. An explanation of the principle of conversion is given in [24]; however, some unverified assumptions were made to simplify the analysis, which, on the other hand, may lead to wrong interpretations about the linearity of the conversion characteristic.

In order to show the linear relationship between $C_{S}$ and *N*, let us consider the RC circuit represented in Figure 2a, where the parameters $R_{\mathrm{RO}}$ and $C_{P}$ were introduced. The parameter $C_{P}$ represents any parasitic capacitance due to the RO and the precharge switch added to the discharge node, while $R_{\mathrm{RO}}$ models the charge absorption rate at each voltage value $V_{C}$. It is important to note that during the full RO cycle, charge is impulsively absorbed due to the sequential switching of the digital gates, causing $V_{C}\left(t\right)$ to resemble a staircase shape. Hence, an effective current $I_{C}$ *per* cycle can be defined, accounting for the amount of charge *q* in the interval of time defined by the *p* period. In our approach, $V_{C}\left(t\right)$ interpolates the actual staircase, allowing for a continuous-time description of the circuit behaviour as in Figure 2b. Hence, $R_{\mathrm{RO}}$ is simply the ratio between the interpolated $V_{C}$ and $I_{C}$. It is convenient to express $R_{\mathrm{RO}}$ and $C_{P}$ as

(2) $$R_{\mathrm{RO}}\left(V_{C}\right)=R_{0}\;u_{\mathrm{RO}}\left(V_{C}\right)\;\mathrm{and}\;C_{P}\left(V_{C}\right)=C_{0}\;u_{P}\left(V_{C}\right),$$

being that $R_{0}=R_{\mathrm{RO}}\left(V_{H}\right)$, $C_{0}=C_{P}\left(V_{H}\right)$ and the functions $u_{\mathrm{RO}}\left(V_{C}\right)$ and $u_{P}\left(V_{C}\right)$ are positive and continuous in the $\left(V_{L},V_{H}\right)$ interval such that $u_{\mathrm{RO}}\left(V_{H}\right)=1$ and $u_{P}\left(V_{H}\right)=1$. The charge absorption rate modelled by $R_{\mathrm{RO}}$ is determined basically by two mechanisms: (i) charge is absorbed due to inter-stage charging within the RO, and (ii) charge is absorbed due to short-circuit currents in the digital gates of the RO at transition times.

The Kirchhoff’s law of currents applied to the simple RC circuit of Figure 2 gives

(3) $$\frac{d\left(\left(C_{S}+C_{P}\right)V_{C}\right)}{dt}=-\frac{V_{C}}{R_{\mathrm{RO}}},$$

where the total charge $Q=\left(C_{S}+C_{P}\right)V_{C}$ is subjected to variations in time due to both $V_{C}\left(t\right)$ and $C_{S}\left(t\right)$, being that the latter is the dynamic component of the capacitive sensor (i.e., the capacitively transduced signal to be converted). This can be neglected when

(4) $$\frac{1}{C_{S}+C_{P}}\left|\frac{dC_{S}}{dt}+\frac{dC_{P}}{dt}\right|\ll \left|\frac{1}{V_{C}}\frac{dV_{C}}{dt}\right|,$$

meaning that at any time point during the conversion, the variations of $C_{S}$ and $C_{P}$ relative to the total capacitance $C_{S}+C_{P}$ are much smaller than the relative variation of $V_{C}$. Such a condition is typically found in a large class of capacitive sensors, where the capacitively transduced signal varies slowly compared to the conversion time $T_{\mathrm{conv}}$. Under this hypothesis, (3) can be simplified in order to obtain

(5) $$\left(1+\frac{C_{0}}{C_{S}}u_{P}\left(V_{C}\right)\right)\;u_{\mathrm{RO}}\left(V_{C}\right)\;\frac{dV_{C}}{V_{C}}=-\frac{dt}{\tau }\;\mathrm{and}\;\tau =R_{0}C_{S}.$$

Note that in a linear RC circuit, i.e., where both $R_{\mathrm{RO}}$ and $C_{P}$ are independent from $V_{C}$, Equation (5) describes the known exponential relaxation of $V_{C}\left(t\right)$, determined by the time-constant τ. The analytical and/or numerical solution of Equation (5) is, in principle, viable once $u_{\mathrm{RO}}\left(V_{C}\right)$ and $u_{P}\left(V_{C}\right)$ are known, either from an analytical insight on a particular RO topology, or directly from fitting simulation data.

The number of counts *N*, stored in dout, is determined by the accumulation of cycles during $T_{\mathrm{conv}}$, which is related to the accumulated phase φ as follows:

(6) $$\varphi \left(T_{\mathrm{conv}}\right)=2\pi \int_{0}^{T_{\mathrm{conv}}}f_{osc}\left(t\right)\;dt\;\mathrm{and}\;N=\lfloor \frac{\varphi (T_{\mathrm{conv}})}{2\pi }\rfloor ,$$

being that $f_{osc}$ is the instantaneous oscillation frequency of p(t). The operator ⌊*x*⌋ indicates the floor operation on the variable *x*. Since $f_{osc}$ is dependent on $V_{C}$, we can elaborate Equation (6) as

(7) $$N=\lfloor \int_{0}^{T_{\mathrm{conv}}}f_{osc}\left(t\right)\;dt\rfloor =\lfloor R_{0}C_{S}\int_{V_{L}}^{V_{H}}\left(1+\frac{C_{0}}{C_{S}}u_{P}\left(V_{C}\right)\right)\;u_{\mathrm{RO}}\left(V_{C}\right)f_{osc}\left(V_{C}\right)\frac{dV_{C}}{V_{C}}\rfloor ,$$

where the differential dt and the time constant τ are substituted with their respective expressions given in Equation (5). For better readability, Equation (7) can be rewritten as

(8) $$N=\lfloor k_{G}C_{S}+k_{G0}C_{0}\rfloor ,$$

where

(9) $$k_{G}=R_{0}\int_{V_{L}}^{V_{H}}\frac{u_{\mathrm{RO}}\left(V_{C}\right)f_{osc}\left(V_{C}\right)}{V_{C}}\;dV_{C};\;k_{G}0=R_{0}\int_{V_{L}}^{V_{H}}\frac{u_{P}\left(V_{C}\right)u_{\mathrm{RO}}\left(V_{C}\right)f_{osc}\left(V_{C}\right)}{V_{C}}\;dV_{C}.$$

The expressions in Equations (8) and (9) remarkably show that *N* is linearly dependent to the input $C_{S}$ through the conversion gain $k_{G}$ regardless of the oscillator implementation, as long as $f_{osc}>0$. An offset term, $k_{G0}C_{0}$, is also present due to any parasitic capacitance added to the precharge node.

The quantization error $\epsilon_{Q}$ is

(10) $$\epsilon_{Q}=\frac{\varphi (T_{\mathrm{conv}})}{2\pi }-N=k_{G}C_{S}+k_{G0}C_{0}-\lfloor k_{G}C_{S}+k_{G0}C_{0}\rfloor .$$

Clear design guidelines can be obtained from the expression of $k_{G}$ of Equation (9) under the following simplifying assumptions. First, let us assume the following relationship between $f_{osc}$ and $V_{C}$, describing the linearised behaviour of the RO:

(11) $$f_{osc}=f_{0}+k_{osc}V_{C},$$

where $f_{0}$ is the frequency bias and $k_{osc}$, given in [s${}^{-1}$V${}^{-1}$], is the voltage sensitivity coefficient. A second simplification regards the $u_{\mathrm{RO}}\left(V_{C}\right)$ function introduced in Equation (2), which is approximated to an effective constant value $u_{\mathrm{RO}}^{⋆}\geq 1$ across the whole interval $\left(V_{L},V_{H}\right)$:

(12) $$u_{\mathrm{RO}}\left(V_{C}\right)=u_{\mathrm{RO}}^{⋆},\;\mathrm{for}\;V_{L}\leq V_{C}\leq V_{H}.$$

Under the assumptions (11)–(12), the integral of Equation (9) is simplified to

(13) $$k_{G}=R_{0}u_{\mathrm{RO}}^{⋆}\cdot \left(f_{0}\log\frac{V_{H}}{V_{L}}+k_{osc}\cdot \left(V_{H}-V_{L}\right)\right).$$

The quantization error referred to as $C_{S}$, i.e., $\varepsilon_{Q}/k_{G}$, is reduced by increasing $k_{G}$ (Equation (10)). Therefore, the simplified expression of $k_{G}$ suggests the following design guidelines:

1. $k_{G}$ is increased by increasing the $R_{0}u_{\mathrm{RO}}^{⋆}$ term, which is related to both the W/L aspect ratio and the area WL of the digital ports and the number of delay stages of the inverter-based RO. The short-circuit current, which contributes to $I_{C}$, is reduced by increasing *L*; however, the short-circuit time interval is minimized by reducing the total area. So for a given gate area WL, it is convenient to reduce the W/L ratio. Clearly, incrementing the number of delay stages increases the discharge rate in each cycle, thus reducing $R_{0}u_{\mathrm{RO}}^{⋆}$.
2. $k_{G}$ is increased by increasing $f_{0}$, which can be attained for minimum-sized transistors, i.e., $W=W_{min}$ and $L=L_{min}$. The parameter $k_{osc}$ depends on the chosen linearisation point, being strongly dependent on the $V_{L}$-$V_{H}$ range. However, as it will be clear in the following discussion, the fully-digital implementation of the CDC rules out this parameter from the design space. As in point 1, reducing the number of delay stages is beneficial to increase $k_{G}$.
3. $k_{G}$ is increased by maximizing $V_{H}$ and minimizing $V_{L}$ as can be seen in the logarithm argument and in the difference term. $V_{H}$ is limited by the available supply voltage value, while $V_{L}$ is limited by the minimum viable supply voltage for the correct operation of the digital gates.

Points 1 and 2, in principle, may lead to divergent design indications as far as the *L* of the digital gates is concerned. For this reason, the optimal solution can be obtained by performing electrical simulations, where *L* is swept across a reasonable interval that includes $L_{min}$.

Regarding the contribution of the comparator physical noise affecting the architecture shown in Figure 2, we can consider the comparator root-mean-square noise $V_{n,\mathrm{cmp}}$. At the end of the conversion, $V_{C}$ will pass the $V_{L}$ threshold with a certain slope, so

(14) $$N_{n,\mathrm{comparator}}≃f_{osc}\left(V_{L}\right)\frac{V_{n,\mathrm{cmp}}}{dV_{C}{/dt|}_{t=T_{\mathrm{conv}}}}≃\tau \;f_{osc}\left(V_{L}\right)u_{\mathrm{RO}}\left(V_{L}\right)\frac{V_{n,\mathrm{cmp}}}{V_{L}},$$

The last part of the approximation is found elaborating Equation (5)—which also gives the definition of τ—and neglecting, for the sake of simplicity, the contribution of $C_{P}$. Equation (14) describes the relationship between the comparator noise and the fluctuation on the conversion code, but most importantly, it establishes also a linear relationship between this fluctuation and the capacitance value through $\tau =R_{0}C_{S}$. This is a very remarkable property of this converter type since the effects of one of the most important sources of physical noise scale proportionally with the quantity to be converted. This also suggests that no particular effort is to be put in the comparator design.

The architecture represented in Figure 2 is based on a continuous-time voltage-domain comparator whose noise effects are analysed in Equation (14). The next step in our analysis is the introduction of the time-domain comparator used in [17], which allows for a fully-digital implementation of the CDC—clearly advantageous since it nulls any static current consumption (except leakage current components).

**Figure 3:**
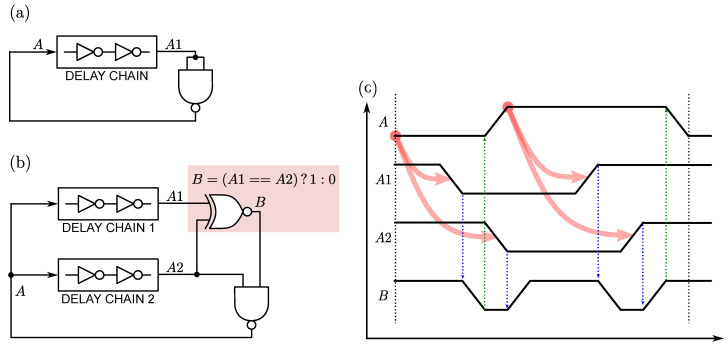
Derivation of a RO with synchronized delay chains: (**a**) starting point representation of a generic RO; (**b**) synchronization principle by a Xnor gate; (**c**) synchronized delay-chain oscillator chronogram.

In order to understand this step, let us consider the synchronized delay-chain RO shown in Figure 3b, derived from the simple RO of Figure 3a. Here, the time-encoded signals, A1 and A2, are originated by two separate delay chains. The following Xnor gate asserts the Boolean “A1==A2” condition, i.e., both signals present the same logic level, so allowing the propagation of the oscillator travelling edge. In a scenario where the travelling edge of A2 lags the one of A1, this assertion permits their synchronization at the Nand gate before closing the feedback loop. Figure 3c shows the chronogram details of the oscillator signals *A*, A1, A2 and *B*.

In the actual CDC operation, A2 is generated by the reference delay chain fed at $V_{L}$, while A1 is generated by the sensing delay chain, fed at $V_{C}$. So, while $V_{C}>V_{L}$, the reference delay chain always lags behind the sensing delay chain. Ideally, both chains are synchronized for $V_{C}=V_{L}$, while the lagging condition is inverted as soon as $V_{C}<V_{L}$, marking the end-of-conversion condition.

**Figure 4:**
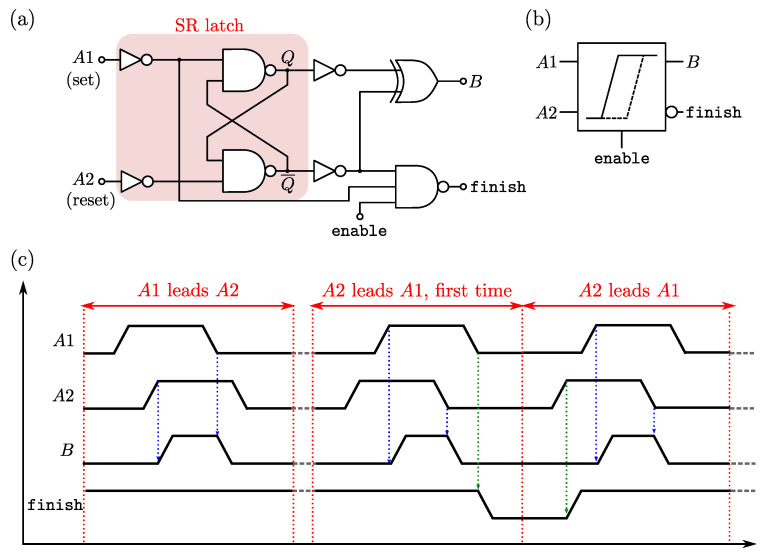
Time-delay comparator: (**a**) schematic diagram, (**b**) symbol view and (**c**) chronogram when operated inside the synchronized delay chains loop.

The time-delay comparator, proposed in [17] and depicted in Figure 4a, provides the same synchronizing function of the Xnor/Nand gates of Figure 3b, while also signalling the end of conversion. It is based on a Nand-type set–reset latch and simple combinational logic to produce the two output signals, *B* and finish. The operation of such circuit is described in Figure 4c considering the following conditions: (i) A1 leads A2, and (ii) A2 leads A1. In both conditions, *B* acts as a synchronization gate, while finish is an active-low signal that pulses only after the first occurrence of the A2-leads-A1 condition. It is important to observe at this point that, while the voltage-domain comparator of Figure 2a is placed outside the RO, the time-domain comparator will be part of the RO, thus contributing to the oscillator parameters, such as $f_{0}$ and the conversion gain $k_{G}$ (see Equation (13)).

**Figure 5:**
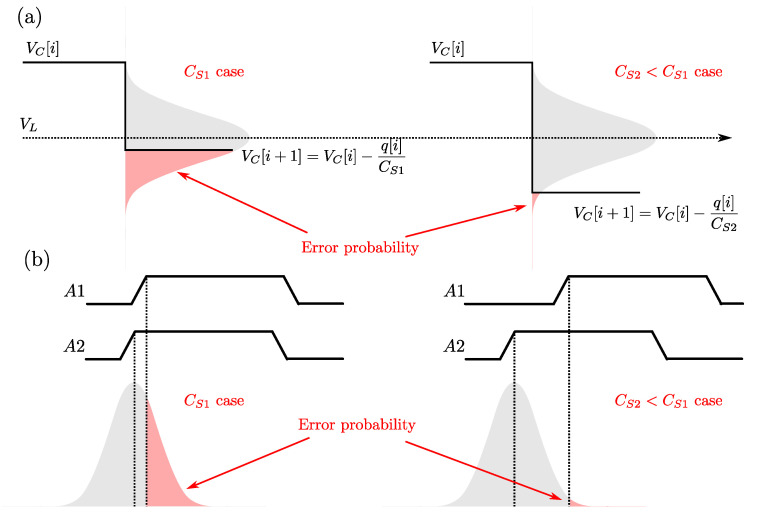
Comparison of comparator noise effects for two different values of $C_{S}$: (**a**) classic voltage-level based comparator as in Figure 2; (**b**) time-delay comparator of Figure 4.

Figure 5 shows the effect of noise on the decision process of the comparator, both voltage-level based and time-delay based, when $V_{C}$ crosses the decision threshold $V_{L}$. The figure shows how a lower value of $C_{S}$ makes the decision process less prone to error since for a constant amount of charge dissipated by the RO in a single cycle, the voltage step (the delay between the travelling edges of A1 and A2) is higher for smaller $C_{S}$ values. This observation is in accordance with Equation (14) and its related discussion on the contribution of comparator noise.

Regarding the rest of noise sources in the circuit, it is well known that a standard voltage-fed RO presents a typical phase-noise spectrum characterized by the $1/f^{3}$ and $1/f^{2}$ behaviours, corresponding to the flicker and thermal noise sources, respectively [25,26,27]. In the synchronized-delay-chains case of Figure 3b, however, part of this noise is rejected due to the synchronization between the travelling edges of A1 and A2. Intuitively, every disturbance (i.e., phase lag or lead) produced after *B* and before *A*, affects both A1 and A2 in the same way, thus showing up as a common-mode noise, rejected by the differential-input nature of the time-delay comparator.

The residual differential-mode phase noise is generated once the RO path is split, corresponding to the separate delay-chain paths before the time-delay comparator. The effects of such noise on the final conversion count are influenced by the interval of time Δt between the A1 and A2 edges. We observe that at the end of conversion, this temporal difference tends to zero; however, the time-domain comparators are less affected by metastability (less prone to error) if the sensitivity of Δt with respect to $V_{C}$, i.e., the quantity $d\left(\Delta t\right)/dV_{C}$, is high.

**Figure 6:**
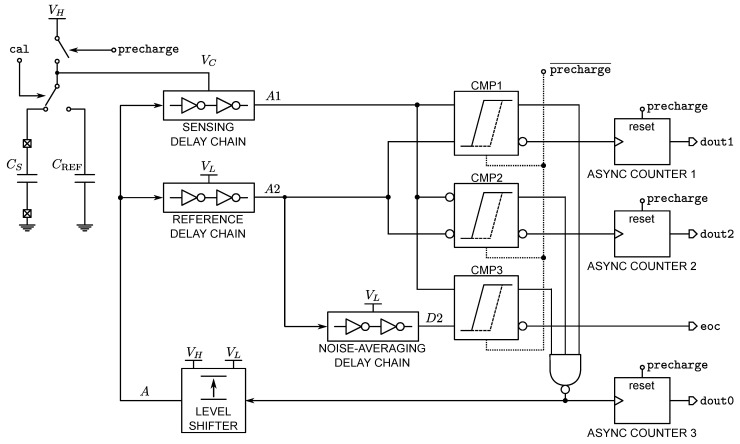
Complete CDC schematic including time-delay comparator-noise averaging and the one-point calibration network.

The complete IDCD-CDC is shown in Figure 6, which features also a noise reduction technique, also proposed in [17], based on correlated averaging on a three-comparators system.

The comparator-noise averaging operates as follows: CMP1 and CMP2 are respectively fed with A1 and A2 and their inverted correspondents, while CMP3 is fed by A1 and a delayed version of A2 (D2). While CMP1’s finish will detect the lagging condition on the rising edges, CMP2’s finish will detect the same condition on the falling edges of A1 and A2. The travelling edges at comparator output are synchronized by a three-input Nand gate. The complete RO loop includes a $V_{L}$-to-$V_{H}$ level shifter that guarantees the correct level transmission to both sensing and reference delay chains. The eoc signal pulses when the A1 travelling edge lags that of D2. Before this condition occurs, the finish outputs of CMP1 and CMP2 have pulsed a certain number of times depending on the amount of extra delay provided by the noise-averaging delay chain. These finish pulses of CMP1 and CMP2 are registered by dedicated counters, which provide dout1 and dout2, respectively. The final conversion code is given by

(15) N=2×dout−(dout1+dout2).

The multiplicative factor of 2 before dout0 accounts for both the rising and falling edges. To give a better understanding of the noise averaging mechanism, let us consider in the first instance that all the delay chains of Figure 6 are identical and their individual delay on the travelling edge dominates over the rest of the elements in the RO, i.e., the time-delay comparators, the Nand gate and the level shifter.

In such a scenario and in absence of noise, if we artificially set $V_{C}=V_{L}$, CMP1 and CMP2 have 50% probability to pulse their finish signals, while CMP3’s finish will not pulse. In order to force CMP3’s finish to pulse, we need to further lower $V_{C}$ to a certain value $V_{C}=V_{L}^{⋆}<V_{L}$. At this point, the conversion ends, meaning that the effective voltage step explored by the sensing chain is $V_{H}-V_{L}^{⋆}$, and thus, some excess count was made. Nevertheless, the finish signals of both CMP1 and CMP2 start to pulse as soon as $V_{C}$ is slightly below $V_{L}$, thus dout1=dout2, accounting for the excess of counts.

When the comparator noise is considered, the probability of CMP1 and CMP2 to make the wrong decision goes from 50% when $V_{C}=V_{L}$ to much lower values, as soon $V_{C}<V_{L}$. By repeating the comparison process a certain number of times at different $V_{C}$ values below $V_{L}$, the probability of error, and thus the noise effect, is reduced. In practice, starting from a certain value of $V_{L}^{⋆}$ far from $V_{L}$, the probability of decision error can be neglected; thus, the decision redundancy only adds up to power consumption. So, in terms of power vs. resolution trade-off, an optimum value $V_{L}^{⋆}$ exists, which can be tuned by the sizing of the noise-averaging delay chain of Figure 6. It must be observed that the crossing of the zone between $V_{L}$ and $V_{L}^{⋆}$ occurs at different slopes, depending of the value of $C_{S}$ to be converted and also depending on $d\left(\Delta t\right)/dV_{C}$, as previously discussed. As a consequence, the number of excess counts increases for higher values of $C_{S}$, having a beneficial effect on the maximum attainable SNR.

The one-point calibration scheme is also shown in Figure 6, implemented through the $C_{\mathrm{REF}}$ capacitance and a switch controlled by the signal cal. The CDC calibration is obtained on demand by operating a conversion on a known value of $C_{\mathrm{REF}}$, obtaining from Equations (6) and (10)

(16) $$N_{\mathrm{REF}}=k_{G}C_{\mathrm{REF}}+k_{G0}C_{0}+\epsilon_{Q,\mathrm{REF}}.$$

The parameters $k_{G}$, $k_{G0}$ and $C_{0}$ may be strongly dependent on process corners and the operating temperature. While the former can be addressed by a one-time calibration at the beginning of the CDC operation, the latter can be addressed by occasionally performing a calibration conversion.

The calibrated value of the conversion, neglecting the physical noise, is obtained by the following formula:

(17) $$C_{S}^{\mathrm{calibrated}}=C_{\mathrm{REF}}\;\frac{N}{N_{\mathrm{REF}}}=C_{S}\;\frac{1+\frac{k_{G0}C_{0}}{k_{G}C_{S}}+\epsilon_{Q}}{1+\frac{k_{G0}C_{0}}{k_{G}C_{\mathrm{REF}}}+\epsilon_{Q,\mathrm{REF}}}.$$

The rightmost side of Equation (17) reveals the residual error after calibration that can be minimized once $C_{S}\gg C_{0}$ and $C_{\mathrm{REF}}\gg C_{0}$ for acceptable quantization errors $\epsilon_{Q}$ and $\epsilon_{Q,\mathrm{REF}}$. Clearly, this calibration method relies profoundly on the stability of the absolute value of $C_{\mathrm{REF}}$. Any process-related dispersion on the nominal value of $C_{\mathrm{REF}}$ affects the conversion value, despite the calibration. From the system-level point of view, two alternative solutions can be adopted. On one side, $C_{\mathrm{REF}}$ can be a very reliable external component, which, however, is affected by connection parasitics. On the other side, $C_{\mathrm{REF}}$ can be integrated all together with the converter circuitry using a metal–oxide–metal (MOM) or a metal–insulator–metal (MIM), when available from the process, capacitor. Nevertheless, the solution concerning the monolithic integration will be affected by the process corners spread. This former hindrance can be overcome by dedicated $C_{\mathrm{REF}}$ testing structures at the wafer level.

### 3.2. 180 nm-CMOS Implementation

Following the design indication explained in Section 3.1, a monolithic implementation of a IDCD-CDC is done in a standard 0.18 µm 1-poly 6-metal-level MIM CMOS technology. In this case study, we aim at optimizing the energy efficiency of the CDC while maintaining 10 effective number of bits (ENOB) of resolution and a total area ≤0.02 mm${}^{2}$. Regarding the operating conditions, we aim for a button-cell operated system; thus, the specification $V_{H}=0.9\;$ V applies for the rest of the discussion.

Referring to Figure 6, all inverters in the delay chain have W=240 nm, L=180 nm. All the delay chains (sensing, reference and noise-averaging) are implemented with 2 stages. With these values, $k_{G}$ results to be 246.468 × 10${}^{-12}$ F${}^{-1}$, and the output code can be stored in a 16-bit output register. The digital gates of CMP1–CMP3, all identical, have all minimal W=240 nm, and L=180 nm.

**Figure 7:**
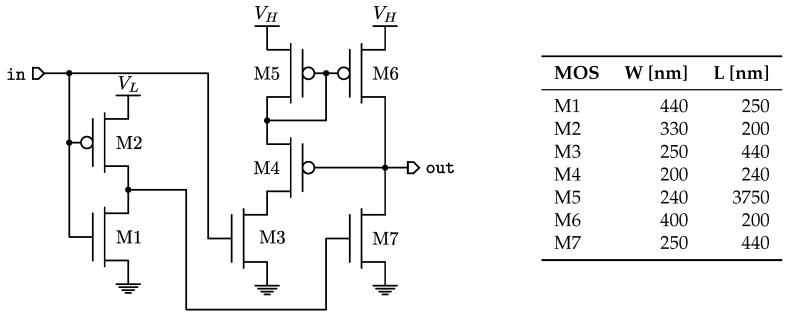
Wilson current-mirror based level shifter and transistor optimized geometrical parameters values for the design case in Section 3.2.

The level shifter topology is adopted from [28]. Its schematic together with the sizes of transistor parameters are shown in Figure 7. Among other possible circuital solutions [29,30,31,32], that of Figure 7 provides the best energy efficiency when operating across subthreshold and super-threshold regions, defined by $V_{L}$ and $V_{H}$. It is important to note that, in this design, the circuit propagation delay is of minor concern since it only affects the conversion time.

The $C_{\mathrm{REF}}$ capacitance is implemented by a MIM capacitor of 10 pF, which is the largest component of the CDC. However, since it is implemented between the two highest top-metal layers, the area underneath is used for the rest of the digital circuitry, using the rest of the metal layers for signal routing.

**Figure 8:**
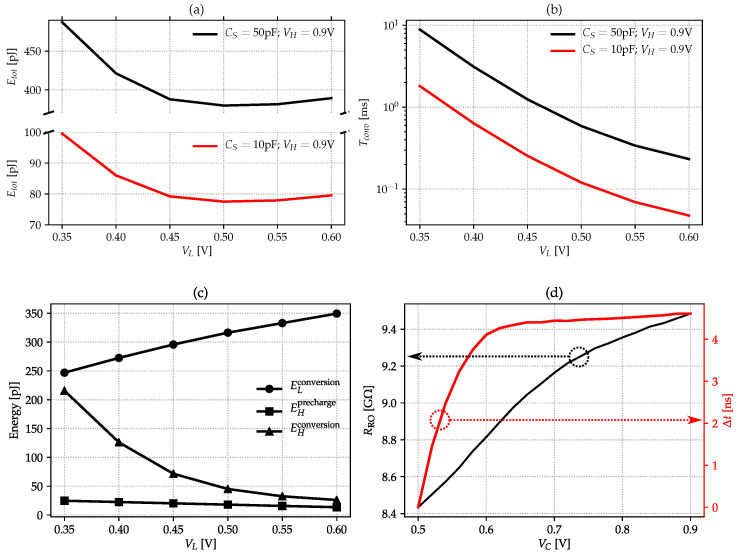
Key design parameters: (**a**) total energy per conversion $E_{tot}$ as function of $V_{L}$ for fixed $V_{H}=0.9$ V; (**b**) energy balance as from Equation (18) as function of $V_{L}$ for $C_{S}=50$ pF and fixed $V_{H}=0.9$ V; (**c**) conversion time $T_{conv}$ as function of $V_{L}$ for fixed $V_{H}=0.9$ V; (**d**) $R_{\mathrm{RO}}$ and Δt as function of $V_{C}$.

The total energy per conversion, $E_{tot}$, and the conversion time, $T_{conv}$, are evaluated as function of $V_{L}$ in order to find an acceptable trade-off between quantization error and energy consumption. Figure 8a shows that a shallow optimum is found for $V_{L}=0.5$ V. This is due to the fact that $E_{tot}$ accounts for currents supplied by the $V_{H}$ and $V_{L}$ sources, respectively $E_{H}$ and $E_{L}$, during the precharge and the conversion phases:

(18) $$E_{tot}=E_{H}^{\mathrm{precharge}}+E_{H}^{\mathrm{conversion}}+E_{L}^{\mathrm{conversion}},$$

where the precharge energy supplied by $V_{H}$ is

(19) $$E_{H}^{\mathrm{precharge}}=C_{S}V_{H}\left(V_{H}-V_{L}\right)$$

and $E_{H}^{\mathrm{conversion}}$ is supplied to the level shifter.

Both Equations (18) and (19) neglect any leakage components, which add up to the total energy balance proportionally to $T_{\mathrm{conv}}$. Equation (19) depicts a monotonically decreasing function of $V_{L}$. The terms $E_{H}^{\mathrm{conversion}}$ and $E_{L}^{\mathrm{conversion}}$, related to the conversion phase, depend on $T_{conv}$, which increase by lowering $V_{L}$, as shown in Figure 8b, where $V_{H}$ is fixed to 0.9 V. Intuitively, we may expect that both $E_{H}^{\mathrm{conversion}}$ and $E_{L}^{\mathrm{conversion}}$ should follow the same trend as $T_{conv}$. This is true for $E_{H}^{\mathrm{conversion}}$, but $E_{L}^{\mathrm{conversion}}$ actually has the opposite behaviour as shown in Figure 8c. This is due to the dominant contribution of comparators activity happening at higher $V_{L}$ values: the higher the $V_{L}$, the higher the $E_{L}^{\mathrm{conversion}}$.

In our design, $V_{L}$ is set to 0.5 V. For such value and for $C_{S}=250$ pF, $E_{tot}=1884\;$fJ, accounting for the following contributions: $E_{H}^{\mathrm{precharge}}=90$ fJ, $E_{H}^{\mathrm{conversion}}=224$ fJ (due to the operation of the level shifter) and $E_{L}^{\mathrm{conversion}}=1570$ fF. The latter is the major contribution since $V_{L}$ supplies also the time-domain comparators and the asynchronous counters.

The behaviour of $R_{\mathrm{RO}}$ as a function of $V_{C}$, introduced in Figure 2a, is shown in Figure 8d along with $\Delta t(V_{C})$. The $R_{\mathrm{RO}}\left(V_{C}\right)$ trend is to increase by increasing $V_{C}$. This is due to the dominant short-circuit currents contributions (transition time shorten as $V_{C}$ increases) over the RO interstage-charging contribution. On the other hand, $\Delta t(V_{C})$ shows a quite noticeable non-linear behaviour. The relatively high value of $d\left(\Delta t\right)/dV_{C}$ in the vicinity of $V_{L}$, resulting to be 71.8 ns/V, favours the CDC immunity against the noise introduced by the split path of the sensing and reference delay chains, as discussed previously.

**Figure 9:**
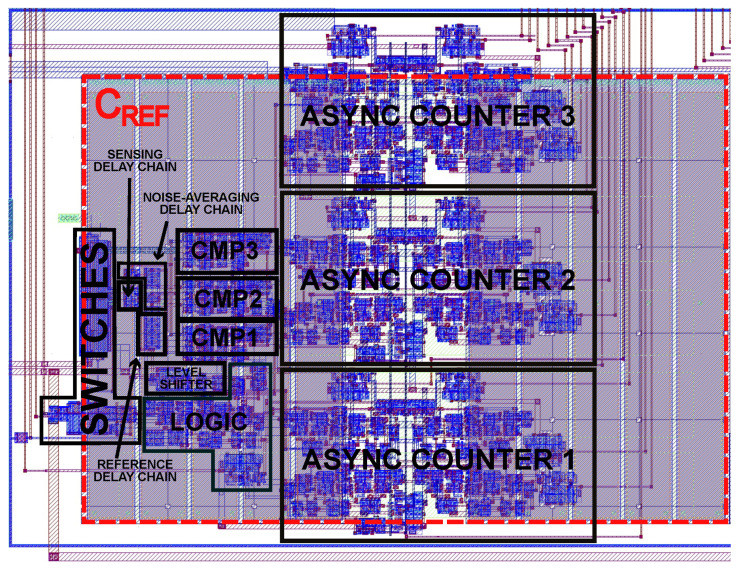
Layout of the CDC in a standard 0.18 µm 1-poly 6-metal-level-MIM CMOS technology. Bounding box size is 160 µm (width) × 120 µm (height).

Finally, the layout of the implemented CDC is shown in Figure 9 showing a silicon area occupancy of 0.0192 mm${}^{2}$ (excluding pads).

### 3.3. Prototype Performance

**Figure 10:**
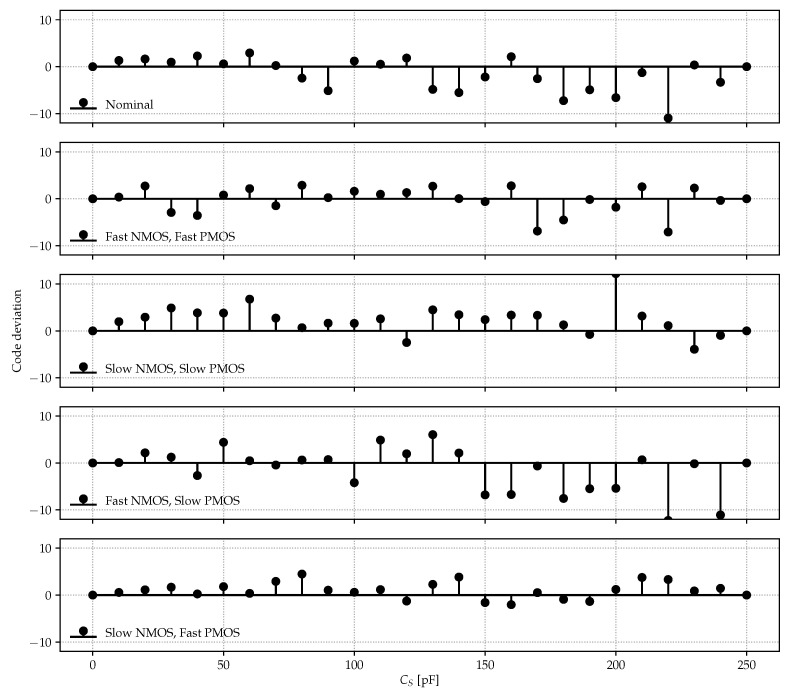
CDC differential non-linearity (DNL) against process corners. The output register width is 16 bits.

The CDC DNL, calculated with respect to the end-points characteristic, when operated at $V_{L}=0.5$ V and $V_{H}=0.9$ V, is shown in Figure 10, and tested against process corners. In all cases, the maximum observed code deviation falls within the ±12 counts interval over an output register of 16 bits, corresponding to an equivalent capacitance LSB of 3.82 fF ($k_{G}=246.468\times 10^{12}\;F^{-1}$). The total energy per conversion scales linearly with $C_{S}$, resulting to be 1.884 nJ at full-scale $C_{S,\mathrm{FS}}=250$ pF. As far as process corner sensitivity is concerned, $E_{tot}$ presents small variations around its nominal value (worst case: +2.9% in the Fast-NMOS Slow-PMOS corner), while at the same time, $T_{conv}$ shows quite large variations: 2.93 ms in the nominal case vs. 0.99 ms and 10.71 ms in the fast-NMOS fast-PMOS and slow-NMOS slow-PMOS, respectively.

The effectiveness of the one-point calibration against process corners is reported in Table 1, where the relative error $\epsilon_{k_{G}}$, defined as

(20) $$\epsilon_{k_{G}}=\frac{k_{G}^{\mathrm{nominal}}-k_{G}}{k_{G}^{\mathrm{nominal}}}$$

is evaluated, showing a ×30 error reduction when calibrated. The systematic offset of the CDC, as in Equation (6), is <255.6 fF. Hence, the CDC shows an input capacitance range from 255.6 fF to 250 pF with a small linearity error of 15.26 ppm.

For the sake of equal comparison, the figure of merit (FoM), as defined in [17], is evaluated:

(21) $$\mathrm{FoM}=\frac{E_{tot}\left(C_{S,\mathrm{FS}}\right)}{2^{(20\log_{10}\left(\mathrm{Input}\;\mathrm{range}/2\sqrt{2}/\mathrm{Resolution}\right)-1.76)/6.02}}=99.61\;\mathrm{fJ}/\mathrm{conversion-step}.$$

where the resolution is calculated only on the basis of nonlinearity effects, while noise is not taken into account.

Transient noise simulations are performed to determine the SNR, which results to be 63.9 dB (10.3 ENOB) at $C_{S,\mathrm{FS}}$. The noise-related FoM, FoM${}_{N}$, of this converter is calculated as

(22) $${\mathrm{FoM}}_{N}=\frac{E_{tot}\left(C_{S,\mathrm{FS}}\right)}{2^{({\mathrm{SNR}}_{\max}-1.76)/6.02}}=1.47\;\mathrm{pJ}/\mathrm{conversion-step}.$$

**Figure 11:**
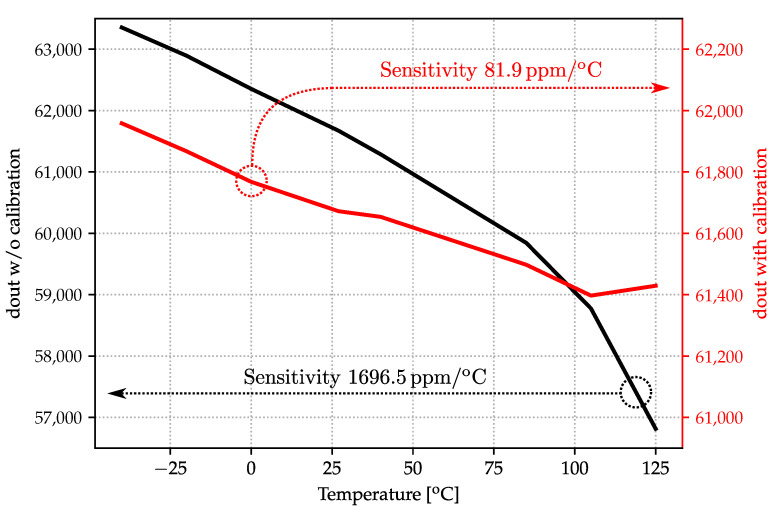
CDC output temperature sensitivity before and after one-point calibration.

Temperature sensitivity is also evaluated as shown in Figure 11, showing a ×20 improvement, from 1696.5 ppm/°C without calibration to 81.9 ppm/°C after calibration, across the −40 °C, +125 °C range.

## 4. Discussion

The CDC based on the IDCD architecture, introduced in [17], has the important characteristic to relay only on digital gates, thus being easily portable among different technological nodes once the fundamental design trade-offs, analysed for the first time in Section 3.1, are taken into account.

Here, we presented a design case, implemented on a low-cost commercial 180 nm-CMOS technology, capable of operating at button-cell supply voltages. Direct comparison with the original implementation of [17] is presented in Table 2. Energy figures are less favourable in the presented design case, as expected, due to the larger minimum feature size of the process used in this work with respect to the case of [17].

The large difference between the FoM and FoM${}_{N}$ values clearly states that, in the current work, distortion effects are much less important than physical noise, while in [17], both distortion and noise contributed to the final resolution of the converter. These aspects confirm the analysis developed in Section 3.1 and give insights into energy efficiency vs. resolution trade-offs of the IDCD-CDC architecture when ported across different CMOS technological nodes.

In conclusion, the IDCD-CDC architecture proves to be a valid solution for capacitive sensor read-out interfaces in the medium/low resolution range. The IDCD-CDC fully exploits the benefits of miniaturization offered by more advanced CMOS technological nodes, while still providing competitive energy figures, even when implemented in low-cost 180 nm CMOS technology. In both cases, compatibility with low-voltage operation is maintained. When looking at evolutions of this architecture, capable of targeting more stringent resolution requirements, the inclusion of additional control circuitry needs to be investigated. Such circuitry should be devoted to the implementation of dynamic techniques for noise reduction and/or noise-shaping mechanisms.

## Figures and Tables

**Table 1:** Conversion-gain relative error $\epsilon_{k_{G}}$ (see Equation (20)) againts process corners: before and after calibration. Offset code, for $C_{S}=0$, is also reported. Nominal offset code is 63.

Process Corner	Uncalibrated $\epsilon_{k_{G}}^{\mathrm{corner}}$ [%]	Calibrated $\epsilon_{k_{G}}^{\mathrm{corner}}$ [%]	Offset Code
Fast NMOS, Fast PMOS	3.126	−0.099	59
Slow NMOS, Slow PMOS	−3.342	0.112	67
Fast NMOS, Slow PMOS	0.437	−0.114	62
Slow NMOS, Fast PMOS	0.213	−0.063	62

**Table 2:** Operative conditions and performance comparison table of IDCD CDCs.

	ISSCC’15 [17]	This Work
Technology	40 nm	180 nm
$V_{H}$, $V_{L}$	1.0 V, 0.45 V	0.9 V, 0.5 V
Input range	0.7 pF to 10 nF	255.6 fF to 250 pF
Linearity error	1090 ppm	15.26 ppm
Conversion time	19.06 µs at $C_{S}=11.3$ pF	132.43 µs at $C_{S}=11.3$ pF 2.93 ms at $C_{S}=250.0$ pF
Conversion energy	35.1 pJ at $C_{S}=11.3$ pF	85.2 pJ at $C_{S}=11.3$ pF 1884.0 pJ at $C_{S}=250.0$ pF
SNR	53.0 dB	63.9 dB
FoM (Equation (21))	141.0 fJ/conversion-step	99.6 fJ/conversion-step
FoM${}_{N}$ (Equation (22))	96.5 fJ/conversion-step	1.47 pJ/conversion-step
Temperature sensitivity	15.5 ppm/°C	81.9 ppm/°C
Core size	42 µm × 40 µm	160 µm × 120 µm including $C_{\mathrm{REF}}$

## Data Availability

Data contained in the text.

## Abbreviations

The following abbreviations are used in this manuscript:

CDC	Capacitance-to-Digital Converter
PM	Phase/Pulse Modulation
ΔΣM	ΔΣ Modulation
CSAR	Capacitive Successive Approximation Register
DR	Dynamic Range
IDCD	Iterative Delay-Chain Discharge
FPCB	Flexible Printed-Circuit Board
PDMS	Polydimethylsiloxane
ASIC	Application-Specific Integrated Circuit
SPI	Serial Peripheral Interface
CMOS	Complementary Metal–Oxide–Semiconductor
MEMS	Micro-Electro-Mechanical Systems
RO	Ring Oscillator
VCO	Voltage-Controlled Oscillator
MOM	Metal–Oxide–Metal
MIM	Metal–Insulator–Metal
SNR	Signal-to-Noise Ratio
ENOB	Effective Number Of Bits
DNL	Differential Non-Linearity
LSB	Least-Significant Bit
FoM	Figure of Merit
